# lncRNA HAR1B has potential to be a predictive marker for pazopanib therapy in patients with sarcoma

**DOI:** 10.3892/ol.2021.12959

**Published:** 2021-08-02

**Authors:** Hideharu Yamada, Masanobu Takahashi, Munenori Watanuki, Mika Watanabe, Sakura Hiraide, Ken Saijo, Keigo Komine, Chikashi Ishioka

Oncol Lett 21: Article no. 455, 2021; DOI: 10.3892/ol.2021.12716

Subsequently to the publication of the above paper, the authors realized that the layouts of Tables I and III had been presented incorrectly, and that they had overlooking acknowledging researchers who had provided them with the cell lines in the Acknowledgements section of the paper.

Consequently, the correctly organized versions of Tables I and 3 are shown on the subsequent pages. Concerning the oversight with the Acknowledgements section, this section of the declarations should be changed to the following (added text is highlighted in bold):

## Figures and Tables

**Table I.** Summary of patient/tumor characteristics and treatment outcomes for 23 patients/tumors analyzed via microarray analyses.

Factors	Total, n (%) (n=23)	Responder, n (%) (n=13)	Non-Responder, n (%) (n=10)
Sex			
Male	14 (61)	7 (54)	7 (70)
Female	9 (39)	6 (46)	3 (30)
Age (years)			
Median	65	65	62
Range	20-76	20-76	31-76
ECOG Performance Status			
0	9 (39)	7 (54)	2 (20)
1	13 (57)	5 (38)	8 (80)
2	1 (4)	1 (8)	0 (0)
>2	0 (0)	0 (0)	0 (0)
Treatment line			
1st line	1 (4)	1 (8)	0 (0)
2nd line	12 (52)	7 (54)	5 (50)
3rd line	7 (30)	3 (23)	4 (40)
4th line	3 (13)	2 (15)	1 (10)
Pathology			
Myxoid LPS	5 (22)	2 (15)	3 (30)
LMS	3 (13)	2 (15)	1 (10)
UPS	3 (13)	2 (15)	1 (10)
SFT	2 (9)	1 (8)	1 (10)
OS	2 (9)	1 (8)	1 (10)
ASPS	2 (9)	2 (15)	0 (0)
US	1 (4)	1 (8)	0 (0)
AS	1 (4)	1 (8)	0 (0)
ES	1 (4)	1 (8)	0 (0)
ESFT	2 (9)	0 (0)	2 (20)
CCS	1 (4)	0 (0)	1 (10)
Primary site			
Extremity	12 (52)	8 (62)	4 (40)
Trunk	2 (9)	1 (8)	1 (10)
Retroperitoneum	1 (4)	1 (8)	0 (0)
Thoracic cavity	1 (4)	1 (8)	0 (0)
Liver	1 (4)	1 (8)	0 (0)
Pancreas	1 (4)	1 (8)	0 (0)
Abdominal cavity	1 (4)	0 (0)	1 (10)
Oral	1 (4)	0 (0)	1 (10)
Pelvis	1 (4)	0 (0)	1 (10)
Sternum	1 (4)	0 (0)	1 (10)
Eye	1 (4)	0 (0)	1 (10)

ECOG, Eastern Cooperative Oncology Group; LPS, liposarcoma; LMS, leiomyosarcoma; UPS, undifferentiated pleomorphic sarcoma; SFT, solitary fibrous tumor; OS, osteosarcoma; ASPS, alveolar soft part sarcoma; US, undifferentiated sarcoma; AS, Angiosarcoma; ES, epithelioid sarcoma; ESFT, Ewing sarcoma family tumor; CCS, clear cell sarcoma.

**Table III.** Functional clusters identified by functional annotation clustering.

A, Cluster number 1, 6 annotation terms included, enrichment score of 2.93			

Top 5 categorized annotation term of each cluster^a^	P-value^b^	Source database^c^	Accession no.
VWC_out	<0.01	SMART	SM00215
VWFC domain	<0.01	InterPro	IPR001007
domain:VWFC 1	<0.01	UniProt	None
domain:VWFC 2	<0.01	UniProt	None
VWC	<0.01	SMART	SM00214

**B, Cluster number 2, 18 annotation terms included, enrichment score of 2.11**			

**Top 5 categorized annotation term of each cluster^a^**	**P-value^b^**	**Source database^c^**	**Accession no.**

Cell membrane	<0.01	UniProt	KW-1003
Signal peptide	<0.01	UniProt	None
Signal	<0.01	UniProt	KW-0732
Glycoprotein	<0.01	UniProt	KW-0325
Disulfide bond	<0.01	UniProt	None

**C, Cluster number 3, 19 annotation terms included, enrichment score of 1.89**			

**Top 5 categorized annotation term of each cluster^a^**	**P-value^b^**	**Source database^c^**	**Accession no.**

EGF-like, conserved site	<0.01	InterPro	IPR013032
EGF-like calcium-binding domain	<0.01	InterPro	IPR001881
EGF-like domain	<0.01	InterPro	IPR000742
EGF_CA	<0.01	SMART	SM00179
domain:EGF-like 4	<0.01	UniProt	None

**D, Cluster number 4, 27 annotation terms included, enrichment score of 1.56**			

**Top 5 categorized annotation term of each cluster^a^**	**P-value^b^**	**Source database^c^**	**Accession no.**

VWC_out	<0.01	SMART	SM00215
VWFC domain	<0.01	InterPro	IPR001007
domain:TIL	<0.01	UniProt	None
domain:VWFD 3	<0.01	UniProt	None
domain:VWFD 2	<0.01	UniProt	None

**E, Cluster number 5, 17 annotation terms included, enrichment score of 1.50**			

**Top 5 categorized annotation term of each cluster^a^**	**P-value^b^**	**Source database^c^**	**Accession no.**

Signaling receptor activity	<0.01	Gene Ontology	GO:0038023
Molecular transducer activity	<0.01	Gene Ontology	GO:0060089
Glycoprotein	<0.01	UniProt	KW-0325
topological domain: Extracellular	<0.01	UniProt	None
Intrinsic component of plasma membrane	<0.01	Gene Ontology	GO:0031226

**F, Cluster number 6, 23 annotation terms included, enrichment score of 1.40**			

**Top 5 categorized annotation term of each cluster^a^**	**P-value^b^**	**Source database^c^**	**Accession no.**

Neurogenesis	<0.01	Gene Ontology	GO:0022008
Neuron differentiation	<0.01	Gene Ontology	GO:0030182
Generation of neurons	<0.01	Gene Ontology	GO:0048699
Cell projection organization	<0.01	Gene Ontology	GO:0030030
Axon development	0.02	Gene Ontology	GO:0061564

**G, Cluster number 7, 12 annotation terms included, enrichment score of 1.33**			

**Top 5 categorized annotation term of each cluster^a^**	**P-value^b^**	**Source database^c^**	**Accession no.**

Inositol phosphate metabolic process	<0.01	Gene Ontology	GO:0043647
Polyol metabolic process	<0.01	Gene Ontology	GO:0019751
Alcohol metabolic process	<0.01	Gene Ontology	GO:0006066
Organic hydroxy compound metabolic process	0.01	Gene Ontology	GO:1901615
Carbohydrate metabolic process	0.04	Gene Ontology	GO:0005975

Seven functional annotation clusters with an enrichment score >1.3 are listed.

a Among each cluster, 5 annotation terms are listed from lower enrichment P-values with its accession no. and source database referred from DAVID (https://david.ncicrf.gov).

b A modified Fisher Exact P-value was generated from gene enrichment analysis.

c Each database is available at the following URLs: SMART, http://smart.embl-heidelberg.de; InterPro, https://www.ebi.ac.uk/interpro; UniProt, https://www.uniprot.org; Gene Ontology, http://geneontology.org. vWF, von Willebrand factor; VWC, von Willebrand factor type C domain; VWFC, von Willebrand factor type C; EGF, epidermal growth factor; EGF_CA, Calcium-binding EGF-like domain; VWFD, von Willebrand factor type D.

## Notes

Note that the above errors did not affect the results or conclusions reported in this paper, and all the authors agree with this corrigendum. The authors thank the editor of *Oncology Letters* for presenting them with the opportunity of publishing this Corrigendum and apologize to the readership of the journal for any inconvenience caused.

